# Supplementary material for: Overlapping cell population expression profiling and regulatory inference in C. elegans
Source: BMC Genomics. 2016 Feb 29;17:159. doi: 10.1186/s12864-016-2482-z (PMC4772325; doi:10.1186/s12864-016-2482-z)
Supplement: Additional file 13: — Web supplement. (DOC 21 kb) [file 12864_2016_2482_MOESM13_ESM.zip › sortWeb/clusters/hier.300.clusters/83.html]

Cluster 83 

## Cluster 83

### Expression

| cnd-1 rep. 1 | cnd-1 rep. 2 | cnd-1 rep. 3 | pha-4 rep. 1 | pha-4 rep. 2 | pha-4 rep. 3 | ceh-27 | ceh-36 | ceh-6 | F21D5.9 | mir-57 | mls-2 | pal-1 | pros-1 | ttx-3 | unc-130 | hlh-16 | irx-1 | ceh-6 (+) hlh-16 (+) | ceh-6 (+) hlh-16 (-) | ceh-6 (-) hlh-16 (+) | cnd-1 singlets | pha-4 singlets | 0 | 60 | 120 | 150 | 180 | 240 | 330 | 390 | 420 | 480 | 540 | 570 | 600 | 630 | 660 | NAME | Functional description |
| --- | --- | --- | --- | --- | --- | --- | --- | --- | --- | --- | --- | --- | --- | --- | --- | --- | --- | --- | --- | --- | --- | --- | --- | --- | --- | --- | --- | --- | --- | --- | --- | --- | --- | --- | --- | --- | --- | --- | --- |
|  |  |  |  |  |  |  |  |  |  |  |  |  |  |  |  |  |  |  |  |  |  |  |  |  |  |  |  |  |  |  |  |  |  |  |  |  |  | *bre-3* | BT (Bacillus thuringiensis) toxin REsistant |
|  |  |  |  |  |  |  |  |  |  |  |  |  |  |  |  |  |  |  |  |  |  |  |  |  |  |  |  |  |  |  |  |  |  |  |  |  |  | *nhr-31* | Nuclear Hormone Receptor family |
|  |  |  |  |  |  |  |  |  |  |  |  |  |  |  |  |  |  |  |  |  |  |  |  |  |  |  |  |  |  |  |  |  |  |  |  |  |  | F23F1.4 |  |
|  |  |  |  |  |  |  |  |  |  |  |  |  |  |  |  |  |  |  |  |  |  |  |  |  |  |  |  |  |  |  |  |  |  |  |  |  |  | *nhr-14* | Nuclear Hormone Receptor family |
|  |  |  |  |  |  |  |  |  |  |  |  |  |  |  |  |  |  |  |  |  |  |  |  |  |  |  |  |  |  |  |  |  |  |  |  |  |  | W03C9.6 |  |
|  |  |  |  |  |  |  |  |  |  |  |  |  |  |  |  |  |  |  |  |  |  |  |  |  |  |  |  |  |  |  |  |  |  |  |  |  |  | K09G1.1 |  |
|  |  |  |  |  |  |  |  |  |  |  |  |  |  |  |  |  |  |  |  |  |  |  |  |  |  |  |  |  |  |  |  |  |  |  |  |  |  | *tbb-6* | TuBulin, Beta |
|  |  |  |  |  |  |  |  |  |  |  |  |  |  |  |  |  |  |  |  |  |  |  |  |  |  |  |  |  |  |  |  |  |  |  |  |  |  | *clec-162* | C-type LECtin |
|  |  |  |  |  |  |  |  |  |  |  |  |  |  |  |  |  |  |  |  |  |  |  |  |  |  |  |  |  |  |  |  |  |  |  |  |  |  | *ttr-34* | TransThyretin-Related family domain |
|  |  |  |  |  |  |  |  |  |  |  |  |  |  |  |  |  |  |  |  |  |  |  |  |  |  |  |  |  |  |  |  |  |  |  |  |  |  | *tag-353* | Temporarily Assigned Gene name |
|  |  |  |  |  |  |  |  |  |  |  |  |  |  |  |  |  |  |  |  |  |  |  |  |  |  |  |  |  |  |  |  |  |  |  |  |  |  | C01F1.5 |  |
|  |  |  |  |  |  |  |  |  |  |  |  |  |  |  |  |  |  |  |  |  |  |  |  |  |  |  |  |  |  |  |  |  |  |  |  |  |  | *cyp-33C7* | CYtochrome P450 family |
|  |  |  |  |  |  |  |  |  |  |  |  |  |  |  |  |  |  |  |  |  |  |  |  |  |  |  |  |  |  |  |  |  |  |  |  |  |  | Y43D4A.8 |  |
|  |  |  |  |  |  |  |  |  |  |  |  |  |  |  |  |  |  |  |  |  |  |  |  |  |  |  |  |  |  |  |  |  |  |  |  |  |  | *suox-1* | SUlfite OXidase |
|  |  |  |  |  |  |  |  |  |  |  |  |  |  |  |  |  |  |  |  |  |  |  |  |  |  |  |  |  |  |  |  |  |  |  |  |  |  | T25C12.7 |  |
|  |  |  |  |  |  |  |  |  |  |  |  |  |  |  |  |  |  |  |  |  |  |  |  |  |  |  |  |  |  |  |  |  |  |  |  |  |  | T25C12.6 |  |
|  |  |  |  |  |  |  |  |  |  |  |  |  |  |  |  |  |  |  |  |  |  |  |  |  |  |  |  |  |  |  |  |  |  |  |  |  |  | *clec-89* | C-type LECtin |
|  |  |  |  |  |  |  |  |  |  |  |  |  |  |  |  |  |  |  |  |  |  |  |  |  |  |  |  |  |  |  |  |  |  |  |  |  |  | *str-94* | Seven TM Receptor |
|  |  |  |  |  |  |  |  |  |  |  |  |  |  |  |  |  |  |  |  |  |  |  |  |  |  |  |  |  |  |  |  |  |  |  |  |  |  | *dpy-19* | DumPY: shorter than wild-type |
|  |  |  |  |  |  |  |  |  |  |  |  |  |  |  |  |  |  |  |  |  |  |  |  |  |  |  |  |  |  |  |  |  |  |  |  |  |  | *best-15* | BESTrophin (chloride channel) homolog |
|  |  |  |  |  |  |  |  |  |  |  |  |  |  |  |  |  |  |  |  |  |  |  |  |  |  |  |  |  |  |  |  |  |  |  |  |  |  | *cka-1* | Choline Kinase A |
|  |  |  |  |  |  |  |  |  |  |  |  |  |  |  |  |  |  |  |  |  |  |  |  |  |  |  |  |  |  |  |  |  |  |  |  |  |  | *rga-5* | Rho GTPase Activating protein |
|  |  |  |  |  |  |  |  |  |  |  |  |  |  |  |  |  |  |  |  |  |  |  |  |  |  |  |  |  |  |  |  |  |  |  |  |  |  | *exc-5* | EXCretory canal abnormal |
|  |  |  |  |  |  |  |  |  |  |  |  |  |  |  |  |  |  |  |  |  |  |  |  |  |  |  |  |  |  |  |  |  |  |  |  |  |  | Y39A1A.27 |  |
|  |  |  |  |  |  |  |  |  |  |  |  |  |  |  |  |  |  |  |  |  |  |  |  |  |  |  |  |  |  |  |  |  |  |  |  |  |  | *toh-1* | TOllisH (tolloid and BMP-1 family) |
|  |  |  |  |  |  |  |  |  |  |  |  |  |  |  |  |  |  |  |  |  |  |  |  |  |  |  |  |  |  |  |  |  |  |  |  |  |  | M03A8.3 |  |
|  |  |  |  |  |  |  |  |  |  |  |  |  |  |  |  |  |  |  |  |  |  |  |  |  |  |  |  |  |  |  |  |  |  |  |  |  |  | F10C1.3 |  |
|  |  |  |  |  |  |  |  |  |  |  |  |  |  |  |  |  |  |  |  |  |  |  |  |  |  |  |  |  |  |  |  |  |  |  |  |  |  | F32F2.2 |  |
|  |  |  |  |  |  |  |  |  |  |  |  |  |  |  |  |  |  |  |  |  |  |  |  |  |  |  |  |  |  |  |  |  |  |  |  |  |  | F16D3.6 |  |
|  |  |  |  |  |  |  |  |  |  |  |  |  |  |  |  |  |  |  |  |  |  |  |  |  |  |  |  |  |  |  |  |  |  |  |  |  |  | F35H8.4 |  |
|  |  |  |  |  |  |  |  |  |  |  |  |  |  |  |  |  |  |  |  |  |  |  |  |  |  |  |  |  |  |  |  |  |  |  |  |  |  | F59E11.7 |  |
|  |  |  |  |  |  |  |  |  |  |  |  |  |  |  |  |  |  |  |  |  |  |  |  |  |  |  |  |  |  |  |  |  |  |  |  |  |  | F36A2.10 |  |
|  |  |  |  |  |  |  |  |  |  |  |  |  |  |  |  |  |  |  |  |  |  |  |  |  |  |  |  |  |  |  |  |  |  |  |  |  |  | K01A2.5 |  |
|  |  |  |  |  |  |  |  |  |  |  |  |  |  |  |  |  |  |  |  |  |  |  |  |  |  |  |  |  |  |  |  |  |  |  |  |  |  | H41C03.1 |  |
|  |  |  |  |  |  |  |  |  |  |  |  |  |  |  |  |  |  |  |  |  |  |  |  |  |  |  |  |  |  |  |  |  |  |  |  |  |  | *lim-6* | LIM domain family |
|  |  |  |  |  |  |  |  |  |  |  |  |  |  |  |  |  |  |  |  |  |  |  |  |  |  |  |  |  |  |  |  |  |  |  |  |  |  | F18C5.9 |  |
|  |  |  |  |  |  |  |  |  |  |  |  |  |  |  |  |  |  |  |  |  |  |  |  |  |  |  |  |  |  |  |  |  |  |  |  |  |  | *lin-3* | abnormal cell LINeage |
|  |  |  |  |  |  |  |  |  |  |  |  |  |  |  |  |  |  |  |  |  |  |  |  |  |  |  |  |  |  |  |  |  |  |  |  |  |  | *adt-1* | ADAMTS family |
|  |  |  |  |  |  |  |  |  |  |  |  |  |  |  |  |  |  |  |  |  |  |  |  |  |  |  |  |  |  |  |  |  |  |  |  |  |  | *dmsr-6* | DroMyoSuppressin Receptor related |
|  |  |  |  |  |  |  |  |  |  |  |  |  |  |  |  |  |  |  |  |  |  |  |  |  |  |  |  |  |  |  |  |  |  |  |  |  |  | *vha-5* | Vacuolar H ATPase |
|  |  |  |  |  |  |  |  |  |  |  |  |  |  |  |  |  |  |  |  |  |  |  |  |  |  |  |  |  |  |  |  |  |  |  |  |  |  | C13C12.2 |  |
|  |  |  |  |  |  |  |  |  |  |  |  |  |  |  |  |  |  |  |  |  |  |  |  |  |  |  |  |  |  |  |  |  |  |  |  |  |  | K08C9.10 |  |
|  |  |  |  |  |  |  |  |  |  |  |  |  |  |  |  |  |  |  |  |  |  |  |  |  |  |  |  |  |  |  |  |  |  |  |  |  |  | *srd-54* | Serpentine Receptor, class D (delta) |
|  |  |  |  |  |  |  |  |  |  |  |  |  |  |  |  |  |  |  |  |  |  |  |  |  |  |  |  |  |  |  |  |  |  |  |  |  |  | R10H1.1 |  |
|  |  |  |  |  |  |  |  |  |  |  |  |  |  |  |  |  |  |  |  |  |  |  |  |  |  |  |  |  |  |  |  |  |  |  |  |  |  | F54B11.11 |  |
|  |  |  |  |  |  |  |  |  |  |  |  |  |  |  |  |  |  |  |  |  |  |  |  |  |  |  |  |  |  |  |  |  |  |  |  |  |  | C44B7.21 |  |
|  |  |  |  |  |  |  |  |  |  |  |  |  |  |  |  |  |  |  |  |  |  |  |  |  |  |  |  |  |  |  |  |  |  |  |  |  |  | *swt-4* | SWEET sugar transporter family |
|  |  |  |  |  |  |  |  |  |  |  |  |  |  |  |  |  |  |  |  |  |  |  |  |  |  |  |  |  |  |  |  |  |  |  |  |  |  | *pfn-2* | ProFiliN |
|  |  |  |  |  |  |  |  |  |  |  |  |  |  |  |  |  |  |  |  |  |  |  |  |  |  |  |  |  |  |  |  |  |  |  |  |  |  | *sqv-7* | SQuashed Vulva |
|  |  |  |  |  |  |  |  |  |  |  |  |  |  |  |  |  |  |  |  |  |  |  |  |  |  |  |  |  |  |  |  |  |  |  |  |  |  | T08G11.2 |  |
|  |  |  |  |  |  |  |  |  |  |  |  |  |  |  |  |  |  |  |  |  |  |  |  |  |  |  |  |  |  |  |  |  |  |  |  |  |  | Y64G10A.354 |  |
|  |  |  |  |  |  |  |  |  |  |  |  |  |  |  |  |  |  |  |  |  |  |  |  |  |  |  |  |  |  |  |  |  |  |  |  |  |  | F35C5.12 |  |
|  |  |  |  |  |  |  |  |  |  |  |  |  |  |  |  |  |  |  |  |  |  |  |  |  |  |  |  |  |  |  |  |  |  |  |  |  |  | *srh-127* | Serpentine Receptor, class H |
|  |  |  |  |  |  |  |  |  |  |  |  |  |  |  |  |  |  |  |  |  |  |  |  |  |  |  |  |  |  |  |  |  |  |  |  |  |  | F26F2.2 |  |
|  |  |  |  |  |  |  |  |  |  |  |  |  |  |  |  |  |  |  |  |  |  |  |  |  |  |  |  |  |  |  |  |  |  |  |  |  |  | F13D11.17 |  |
|  |  |  |  |  |  |  |  |  |  |  |  |  |  |  |  |  |  |  |  |  |  |  |  |  |  |  |  |  |  |  |  |  |  |  |  |  |  | H05C05.4 |  |
|  |  |  |  |  |  |  |  |  |  |  |  |  |  |  |  |  |  |  |  |  |  |  |  |  |  |  |  |  |  |  |  |  |  |  |  |  |  | K09B3.1 |  |
|  |  |  |  |  |  |  |  |  |  |  |  |  |  |  |  |  |  |  |  |  |  |  |  |  |  |  |  |  |  |  |  |  |  |  |  |  |  | T07G12.2 |  |
|  |  |  |  |  |  |  |  |  |  |  |  |  |  |  |  |  |  |  |  |  |  |  |  |  |  |  |  |  |  |  |  |  |  |  |  |  |  | *gst-23* | Glutathione S-Transferase |
|  |  |  |  |  |  |  |  |  |  |  |  |  |  |  |  |  |  |  |  |  |  |  |  |  |  |  |  |  |  |  |  |  |  |  |  |  |  | *linc-68* | Long Intervening Non-Coding RNA |
|  |  |  |  |  |  |  |  |  |  |  |  |  |  |  |  |  |  |  |  |  |  |  |  |  |  |  |  |  |  |  |  |  |  |  |  |  |  | F40H3.2 |  |

### Phenotypes enriched

none found

### Anatomy terms enriched

none found

### GO terms enriched

none found

### Expression clusters enriched

none found

### Motifs enriched

|  |  |  |  |  |  |
| --- | --- | --- | --- | --- | --- |
| **Motif** | **Logo** | **Possible orthologs** | **Number of motifs in cluster** | **Enrichment** | **FDR corrected p** |
| pTH6636 |  | egl-5 | 23 | 3.48 | 1.5e-05 |
| Hr78\_SANGER\_5\_FBgn0015239 |  | nhr-19 nhr-213 | 26 | 3.08 | 1.5e-05 |
| RORG\_f1 |  | nhr-213 | 58 | 1.34 | 5.0e-04 |
| EN2\_2 |  | ceh-1 (0.64) ceh-16 ceh-31 | 35 | 2.01 | 5.0e-04 |
| pTH9163 |  | nhr-3 | 16 | 3.64 | 8.9e-04 |
| ftz-f1\_SANGER\_5\_FBgn0001078 |  | nhr-2 (-0.54) nhr-10 nhr-68 nhr-6 nhr-213 nhr-71 | 25 | 2.43 | 1.3e-03 |
| Slou\_Cell\_FBgn0002941 |  | ceh-1 (0.64) lim-7 ceh-30 ceh-9 ceh-24 ceh-31 | 40 | 1.72 | 2.0e-03 |
| FOXJ3\_3 |  | daf-16 let-381 lin-31 fkh-7 pha-4 | 21 | 2.64 | 2.5e-03 |
| pTH6195 |  | ceh-1 (0.64) ceh-16 lim-7 egl-5 cog-1 ceh-10 ceh-30 ceh-14 alr-1 ceh-12 ceh-45 ceh-2 ceh-43 pha-2 lin-39 ceh-31 | 36 | 1.83 | 2.5e-03 |
| pTH9237 |  | mel-28 (-0.55) | 57 | 1.31 | 2.7e-03 |
| V$XFD3\_01 |  | let-381 | 41 | 1.67 | 2.7e-03 |
| FOXO1\_si |  | daf-16 | 31 | 2.00 | 2.7e-03 |
| Isx\_3445 |  | ceh-1 (0.64) alr-1 ceh-12 ceh-45 ceh-43 | 36 | 1.81 | 2.8e-03 |
| pTH10807 |  | nhr-86 (-0.7) F13H6.1 nhr-177 | 22 | 2.49 | 3.4e-03 |
| FOXI1\_2 |  | daf-16 lin-31 fkh-7 | 55 | 1.35 | 3.5e-03 |
| pTH10779 |  | nhr-134 nhr-182 | 13 | 3.83 | 3.6e-03 |
| GRHL1\_1 |  | grh-1 (0.8) | 18 | 2.85 | 4.0e-03 |
| pTH9322 |  | nhr-10 nhr-7 elt-1 nhr-100 | 33 | 1.88 | 4.2e-03 |
| pTH3712 |  | D1081.8 | 10 | 4.83 | 4.7e-03 |
| pTH6106 |  | nhr-182 | 7 | 7.14 | 6.5e-03 |
| pTH10647 |  | nhr-232 | 15 | 3.14 | 6.6e-03 |
| Nkx1-1\_3856 |  | ceh-30 | 23 | 2.29 | 6.6e-03 |
| pTH10714 |  | nhr-142 nhr-84 | 10 | 4.58 | 6.8e-03 |
| V$FAC1\_01 |  | gei-8 | 41 | 1.60 | 7.1e-03 |
| Mw160 |  | nhr-68 | 31 | 1.89 | 7.1e-03 |
| I$ELF1\_01 |  | grh-1 (0.8) sox-4 dmd-4 | 42 | 1.57 | 7.7e-03 |
| MA0164.1 |  | nhr-100 | 52 | 1.37 | 7.8e-03 |
| V$TCF11\_01 |  | skn-1 | 57 | 1.27 | 8.2e-03 |
| Mw145 |  | elt-1 | 61 | 1.17 | 8.2e-03 |
| MA0508.1 |  | blmp-1 (0.78) | 24 | 2.18 | 8.5e-03 |
| pTH9924 |  | nhr-46 | 24 | 2.16 | 9.4e-03 |
| V$S8\_01 |  | ceh-45 | 34 | 1.75 | 9.8e-03 |
| En2\_0952 |  | ceh-16 | 37 | 1.66 | 1.1e-02 |
| Ubx\_FlyReg\_FBgn0003944 |  | alr-1 lin-39 | 55 | 1.30 | 1.1e-02 |
| V$BRACH\_01 |  | tbx-39 mab-9 | 20 | 2.40 | 1.1e-02 |
| Hmbox1\_2674 |  | hmbx-1 | 20 | 2.40 | 1.1e-02 |
| FEV\_f1 |  | lin-1 | 24 | 2.13 | 1.1e-02 |
| pTH8318 |  | attf-1 | 11 | 3.84 | 1.2e-02 |
| FOXD3\_1 |  | let-381 ceh-43 | 13 | 3.30 | 1.2e-02 |
| SRP000217\_Sox2 |  | ceh-18 sox-4 tbp-1 | 45 | 1.48 | 1.2e-02 |
| BARHL2\_4 |  | ceh-31 | 12 | 3.52 | 1.2e-02 |
| PAX5\_f1 |  | pax-2 | 21 | 2.30 | 1.3e-02 |
| Irx6\_2623 |  | irx-1 | 56 | 1.28 | 1.3e-02 |
| FOXC1\_1 |  | let-381 lin-31 | 30 | 1.85 | 1.3e-02 |
| pTH10823 |  | B0310.2 | 39 | 1.60 | 1.3e-02 |
| BARHL2\_6 |  | ceh-31 | 13 | 3.24 | 1.3e-02 |
| Foxj3\_1 |  | lin-31 | 24 | 2.10 | 1.4e-02 |
| Sox21\_3417 |  | pop-1 sox-4 C05C9.3 | 38 | 1.62 | 1.4e-02 |
| NR2F6\_f1 |  | nhr-2 (-0.54) nhr-62 | 32 | 1.78 | 1.4e-02 |
| pTH9085 |  | nhr-42 | 8 | 5.17 | 1.4e-02 |
| CG8765\_SANGER\_5\_FBgn0036900 |  | H20J04.3 | 33 | 1.74 | 1.5e-02 |
| V$LYF1\_01 |  | F26F4.8 (-0.52) mbr-1 | 33 | 1.74 | 1.5e-02 |
| pTH9907 |  | nhr-34 (0.64) | 22 | 2.20 | 1.5e-02 |
| MA0244.1 |  | C48E7.11 | 36 | 1.66 | 1.5e-02 |
| V$HOX13\_01 |  | lin-39 | 31 | 1.80 | 1.6e-02 |
| pTH9072 |  | klf-1 | 50 | 1.37 | 1.6e-02 |
| Lhx1\_2240 |  | lim-7 | 32 | 1.75 | 1.7e-02 |
| V$EGR2\_01 |  | ZC328.2 klf-2 | 9 | 4.37 | 1.8e-02 |
| pTH9182 |  | tbx-39 | 36 | 1.64 | 1.8e-02 |
| pTH10013 |  | nhr-168 (0.51) | 13 | 3.12 | 1.8e-02 |
| pTH8983 |  | tag-347 | 19 | 2.37 | 1.8e-02 |
| V$GATA1\_06 |  | elt-1 | 14 | 2.92 | 1.9e-02 |
| V$CART1\_01 |  | alr-1 | 20 | 2.27 | 2.0e-02 |
| Scr\_Cell\_FBgn0003339 |  | lin-39 | 37 | 1.60 | 2.1e-02 |
| Sox15\_3457 |  | sox-4 | 41 | 1.52 | 2.1e-02 |
| NR2E3\_f1 |  | nhr-100 | 32 | 1.73 | 2.1e-02 |
| Vsx1\_1728 |  | alr-1 | 35 | 1.65 | 2.2e-02 |
| NR2F6\_2 |  | nhr-2 (-0.54) nhr-62 | 22 | 2.12 | 2.2e-02 |
| Hlxb9\_3422 |  | ceh-12 | 27 | 1.89 | 2.2e-02 |
| pTH8998 |  | mab-3 | 14 | 2.85 | 2.3e-02 |
| pTH3997 |  | C04F5.9 | 49 | 1.37 | 2.3e-02 |
| I$KR\_01 |  | B0310.2 | 13 | 3.00 | 2.4e-02 |
| HXA10\_f1 |  | lin-39 | 37 | 1.59 | 2.4e-02 |
| Tcf2\_0913 |  | hmbx-1 | 18 | 2.38 | 2.4e-02 |
| Cart1\_1275 |  | lim-7 alr-1 | 36 | 1.61 | 2.5e-02 |
| pTH8991 |  | cey-3 (-0.54) | 18 | 2.37 | 2.5e-02 |
| pTH9247 |  | C34D1.1 | 36 | 1.60 | 2.6e-02 |
| CG5669\_SANGER\_10\_FBgn0039169 |  | klf-2 klf-1 | 13 | 2.96 | 2.7e-02 |
| MA0488.1 |  | crh-1 (-0.54) | 41 | 1.50 | 2.7e-02 |
| HXD13\_f1 |  | pal-1 | 38 | 1.55 | 2.8e-02 |
| MA0095.2 |  | lsy-2 (-0.52) | 23 | 2.02 | 2.8e-02 |
| MA0131.1 |  | F39B2.1 (-0.63) | 16 | 2.50 | 3.1e-02 |
| PITX2\_si |  | ceh-53 | 17 | 2.40 | 3.1e-02 |
| ELF3\_f1 |  | C24A1.2 | 13 | 2.88 | 3.2e-02 |
| MA0599.1 |  | klf-2 klf-1 | 32 | 1.68 | 3.2e-02 |
| pTH9323 |  | odr-7 | 33 | 1.65 | 3.3e-02 |
| nau\_da\_SANGER\_5\_FBgn0002922 |  | hlh-1 | 27 | 1.82 | 3.4e-02 |
| pTH5561 |  | nhr-239 | 22 | 2.04 | 3.4e-02 |
| Dlx2\_2273 |  | ceh-43 | 30 | 1.73 | 3.5e-02 |
| MA0126.1 |  | lin-48 pax-3 | 38 | 1.53 | 3.5e-02 |
| TBX2\_f1 |  | tbx-39 | 35 | 1.60 | 3.5e-02 |
| En1\_3123 |  | ceh-16 | 58 | 1.20 | 3.6e-02 |
| CG31670\_SOLEXA\_5\_FBgn0031375 |  | CELE\_Y38H8A.5 | 43 | 1.44 | 3.6e-02 |
| MEF2D\_f1 |  | mef-2 | 34 | 1.62 | 3.7e-02 |
| MA0247.2 |  | ceh-22 | 55 | 1.25 | 3.8e-02 |
| V$FREAC7\_01 |  | lin-31 | 56 | 1.23 | 3.9e-02 |
| ONEC2\_si |  | ceh-48 (-0.51) | 29 | 1.74 | 3.9e-02 |
| Nkx2-5\_3436 |  | ceh-24 | 52 | 1.29 | 3.9e-02 |
| Fkh2 |  | fkh-10 | 23 | 1.96 | 3.9e-02 |
| pTH9928 |  | jun-1 | 39 | 1.50 | 4.0e-02 |
| MYF6\_1 |  | hlh-1 | 23 | 1.95 | 4.2e-02 |
| GM12878\_SRF\_HudsonAlpha |  | unc-120 | 39 | 1.50 | 4.3e-02 |
| ETV3\_1 |  | lin-1 | 23 | 1.94 | 4.5e-02 |
| pTH6268 |  | ceh-2 | 11 | 3.10 | 4.5e-02 |
| pTH3041 |  | atf-2 | 52 | 1.29 | 4.6e-02 |
| Nkx1-2\_3214 |  | ceh-30 | 25 | 1.85 | 4.6e-02 |
| FOXI1\_f1 |  | lin-31 | 34 | 1.59 | 4.6e-02 |
| pTH10038 |  | gei-3 | 32 | 1.64 | 4.6e-02 |
| pTH9974 |  | hlh-16 | 58 | 1.19 | 4.6e-02 |
| Sox17\_2837 |  | sox-4 | 44 | 1.41 | 4.7e-02 |
| Sox4 |  | pop-1 | 36 | 1.55 | 4.7e-02 |
| Hoxd1\_3448 |  | ceh-12 | 29 | 1.72 | 4.7e-02 |
| CG2052\_SOLEXA\_2.5\_FBgn0039905 |  | fkh-7 | 24 | 1.89 | 4.8e-02 |
| Cutl1\_3494 |  | ceh-44 (-0.55) | 44 | 1.40 | 4.8e-02 |
| pTH6641 |  | lin-31 | 26 | 1.80 | 5.0e-02 |

### Correlated (and anti-correlated) transcription factors

|  |  |
| --- | --- |
| **Transcription factor** | **Correlation** |
| nhr-14 | 0.85 |
| nhr-146 | 0.84 |
| nhr-31 | 0.81 |
| grh-1 | 0.80 |
| atf-8 | 0.79 |
| blmp-1 | 0.78 |
| nhr-87 | 0.78 |
| nhr-104 | 0.75 |
| dhhc-2 | 0.72 |
| nhr-204 | 0.71 |
| ztf-27 | 0.69 |
| madf-1 | 0.69 |
| bed-3 | 0.67 |
| nhr-92 | 0.67 |
| nhr-5 | 0.66 |
| nhr-23 | 0.64 |
| nhr-34 | 0.64 |
| nhr-112 | 0.64 |
| lim-6 | 0.64 |
| ceh-1 | 0.64 |
| fkh-9 | 0.63 |
| pros-1 | 0.63 |
| mxl-3 | 0.60 |
| nhr-70 | 0.59 |
| gmeb-3 | 0.58 |
| rnf-113 | -0.68 |
| ztf-18 | -0.68 |
| F13C5.2 | -0.68 |
| R151.8 | -0.69 |
| F23A7.6 | -0.69 |
| atf-7 | -0.69 |
| madf-7 | -0.70 |
| nhr-86 | -0.70 |
| snpc-4 | -0.70 |
| T11G6.8 | -0.71 |
| bed-1 | -0.71 |
| dpl-1 | -0.72 |
| F17C11.1 | -0.72 |
| efl-1 | -0.73 |
| sdc-1 | -0.73 |
| ceh-91 | -0.74 |
| Y48G8AL.9 | -0.74 |
| eor-1 | -0.74 |
| lir-2 | -0.75 |
| nfyb-1 | -0.76 |
| isw-1 | -0.76 |
| wrm-1 | -0.78 |
| aptf-2 | -0.78 |
| hmg-1.1 | -0.79 |
| T22C8.3 | -0.84 |

### ChIP peaks enriched

|  |  |  |  |  |
| --- | --- | --- | --- | --- |
| **Gene** | **Experiment** | **Number of upstream peaks** | **Enrichment** | **FDR corrected p** |
| nhr-77 | NHR-77\_Larvae-L4-stage | 17 | 2.68 | 0.0047 |
| F45C12.2 | F45C12.2\_Larvae-L3-stage | 3 | 26.27 | 0.0081 |
| hpl-2 | HPL-2\_Fed-L1-stage-larvae | 15 | 2.40 | 0.0340 |
| sax-3 | SAX-3\_Larvae-L3-stage | 11 | 2.82 | 0.0480 |
